# Hospitalizations for Chronic Obstructive Pulmonary Disease Exacerbation During COVID-19

**DOI:** 10.1001/jamanetworkopen.2024.12383

**Published:** 2024-05-21

**Authors:** Arnaud Bourdin, Engi Ahmed, Isabelle Vachier, Nicolas Roche, Joana Pissarra, Nicolas Malafaye, Nicolas Molinari

**Affiliations:** 1 Department of Respiratory Diseases, University of Montpellier, Centre Hospitalier Universitaire (CHU) Montpellier, Montpellier, France; 2 Laboratoire de Physiologie et Médecine Expérimentale du Cœur et des Muscles, University of Montpellier, Centre National de la Recherche Scientifique, Institut National de la Santé et de la Recherche Médicale (INSERM), CHU Montpellier, Montpellier, France; 3 Laboratory of Immunoregulation and Mucosal Immunology, VIB-UGent Center for Inflammation Research, Ghent, Belgium; 4 Assistance Publique–Hôpitaux de Paris, Centre–Université Paris Cité, Cochin Hospital and Institute (INSERM UMR1016), Respiratory Medicine, Paris, France; 5 Clinical Research and Epidemiology Unit, CHU Montpellier, University of Montpellier, Montpellier, France; 6 Institut Desbrest d’Épidémiologie et de Santé Publique, INSERM, Precision Medicine by Data Integration and Causal Learning, Institut National de Recherche en Informatique et en Automatique, University of Montpellier, CHU Montpellier, Montpellier, France

## Abstract

This cross-sectional study investigates changes in the number of chronic obstructive pulmonary disease (COPD)–related admissions before, during, and after the COVID-19 pandemic in France.

## Introduction

The extent to which airborne infections trigger chronic obstructive pulmonary disease (COPD) exacerbations is debated.^1^ Bacteria, air pollution, heart failure, and other causes worsen COPD symptoms and precipitate unscheduled medical care. Extensive data show that bacterial and viral infections stimulate inflammation, thereby inducing exacerbations.^2,3^ Seasonal peaks are a hallmark characteristic of COPD exacerbations and viral infections, such as influenza or colds, further supporting their association.^4^

Clinicians and patients experienced this seasonality until 2020, when the COVID-19 pandemic began. Remembering the possible contribution of respiratory viruses to onset of COPD exacerbations, we investigated the number of COPD-related admissions before, during, and after the COVID-19 pandemic.

## Methods

This retrospective cross-sectional study is based on French National Health Data System data (eMethods in Supplement 1). An algorithm based on *International Statistical Classification of Diseases and Related Health Problems, Tenth Revision* (*ICD-10*) codes extracted COPD exacerbations in any hospital admission in France from January 1, 2013, to July 31, 2023. An interrupted time series analysis (ITSA) used the R package its.analysis, version 4.3.1, to model the association of preventive measures with COPD admissions and linear regression for graphic representation only. According to French law, this anonymous retrospective observational database study did not require ethics committee approval or patient informed consent. We followed the STROBE reporting guideline. Two-sided statistical tests indicated significance at *P* < .05.

## Results

Of 800 730 patients (1 393 825 admissions), mean (SD) age was 74.4 (12.5) years; 62.5% were male and 37.5 were female. Prevalence of relevant comorbidities included 63.5% hypertension, 43.2% heart failure, 6.9% lung cancer, and 2.4% bladder cancer. Prior to COVID-19, the seasonality of hospital and intensive care unit stays for COPD exacerbations showed a distinct pattern, with single or double peaks during fall and winter (Figure). In March 2020, COPD exacerbations plummeted at the initiation of COVID-19 lockdowns and remained low until late 2021. This reduction occurred immediately after the introduction of infection transmission protective measures (social distancing, masks, and extensive hand hygiene). In 2022, viral transmission preventive measures were less strict. An increasing but still reduced number of COPD exacerbations was recorded in June and July and winter of 2022. Once the World Health Organization declared the pandemic over in May 2023 and people abandoned widespread infection prevention measures, the seasonal peak in COPD exacerbations returned to its usual magnitude. The ITSA confirmed these observations and showed significant variation between periods for severe (*F* = 5.081; *P* = .008) and mild (*F* = 3.850; *P* = .02) cases (Table).

**Figure.**
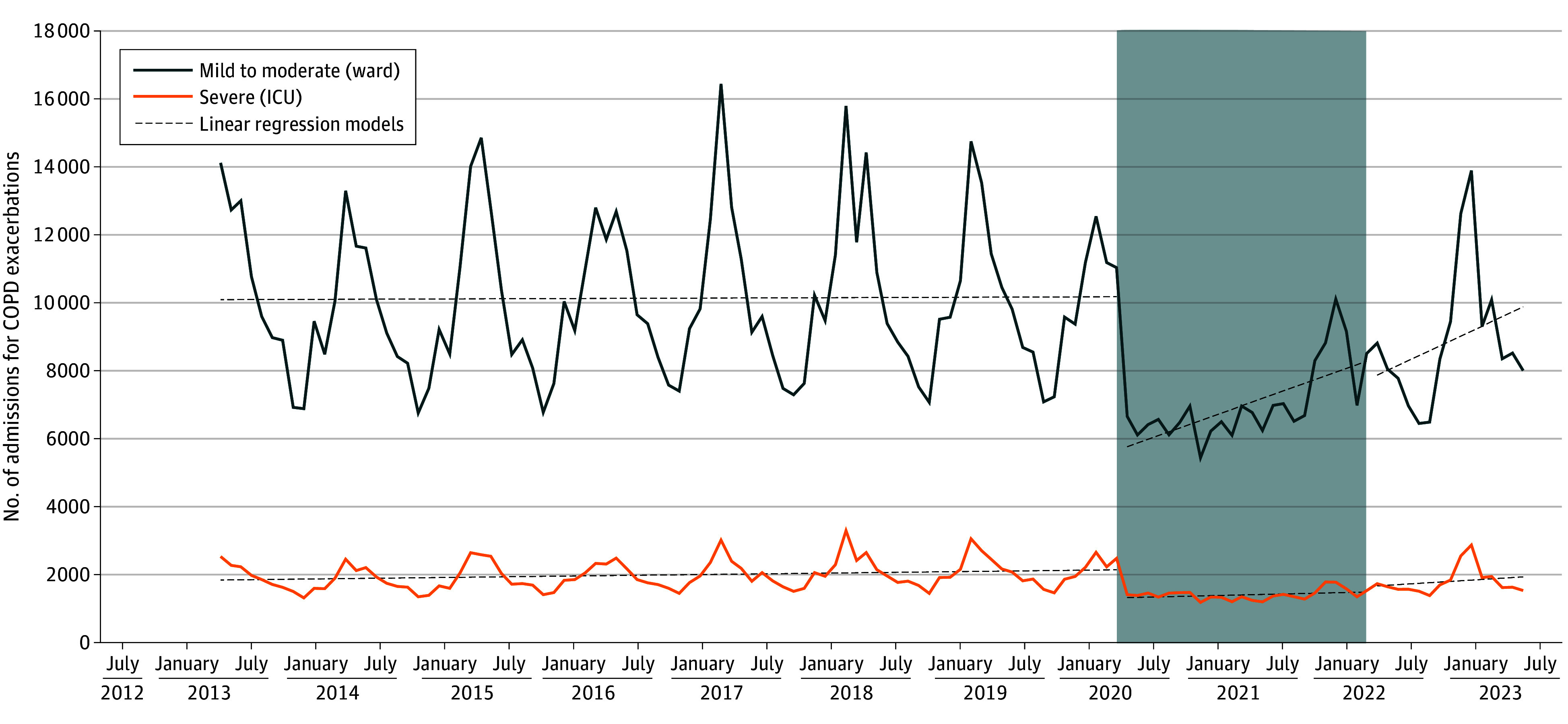
Nationwide Number of Admissions for Chronic Obstructive Pulmonary Disease (COPD) Exacerbations Gray shading indicates the official beginning and end of the social distancing measures enforcement. Dashed lines indicate the linear trend for each period (before, during, and after implementation of preventive measures due to the COVID-19 pandemic). ICU indicates intensive care unit.

**Table.** COPD Admissions by Severity and ITSA Results

Case severity	COPD admissions, mean (SD)			ITSA modeling output			
	Before COVID-19^a^	During COVID-19^b^	After COVID-19^c^	Sum of squares	*F* value	*P* value	Bootstrapped *F* value (95% CI)
Mild	10 122 (2256)	7010 (1127)	8862 (2068)	17 922 979	3.850	.02	4.231 (1.129-10.682)
Severe	1971 (411)	1385 (154)	1780 (404)	828 753	5.081	.008	5.524 (1.696-13.403)

Abbreviations: COPD, chronic obstructive pulmonary disease; ITSA, interrupted time series analysis.

^a^ From January 1, 2013, to March 15, 2020.

^b^ From March 16, 2020, to March 15, 2022.

^c^ From March 16, 2022, to July 31, 2023.

## Discussion

The association between COPD exacerbations and preventive measures questions the role airborne infections play in provoking COPD exacerbations. Other known risk factors were also decreased during the pandemic (eg, small particulate matter levels were reduced^5^), and a single underlying cause behind COPD exacerbations is unlikely. Nevertheless, the “on-off-on” outcome of protective measures showed how epidemiology can provide evidence mimicking a gene knockout–rescue model. Patients with COPD reported strongly adhering to such measures,^6^ thereby reducing their exposure to airborne viruses. These data show that the implementation of infection transmission preventive measures overlaps the drop in COPD exacerbations, and the recirculation of seasonal viruses matches the return of COPD exacerbations, corroborating the involvement of viral infection in COPD exacerbations.

Study limitations include the lack of individual clinical information in the national registry and no direct comparison with the number of diagnosed viral infections. Relevant data on demographic characteristics and comorbidities confirm that the patients’ profiles align with what is expected in a population with COPD.

These data support the use of individual protective measures during winter by patients with COPD and their relatives and caregivers. The graph line (Figure) becomes a starting point for describing the underlying mechanisms behind exacerbations and their relative contributions, and the debate around viral infections triggering COPD exacerbations may be finally settled.

## Notes

Supplement 1. **eMethods.** Data Accrual and Analysis
eReferences

Supplement 2.
Data Sharing Statement
