# Supplementary material for: Hospitalizations for Chronic Obstructive Pulmonary Disease Exacerbation During COVID-19
Source: JAMA Netw Open. 2024 May 21;7(5):e2412383. doi: 10.1001/jamanetworkopen.2024.12383 (PMC11109769; doi:10.1001/jamanetworkopen.2024.12383)
Supplement: Supplement 1. — eMethods. Data Accrual and Analysis eReferences [file jamanetwopen-e2412383-s001.pdf]

Supplemental Online Content

Bourdin A, Ahmed E, Vachier I, et al. Hospitalizations for chronic obstructive pulmonary disease exacerbation during COVID-19. *JAMA Netw Open*. 2024;7(5):e2412383. doi:10.1001/jamanetworkopen.2024.12383

eMethods. Data Accrual and Analysis

eReferences

This supplemental material has been provided by the authors to give readers additional information about their work.

© 2024 Bourdin A et al. *JAMA Network Open.*

Methods. Data Accrual and Analysis

This is a retrospective observational study based on COPD admissions data from January 2012 to July 2023 collected from the national French registry SNDS, an excellent source of reliable epidemiological data1[1]. The SNDS (*Système National Des Données De Santé*) merges data from five different databases and is one of the most comprehensive health databases worldwide (https://www.snds.gouv.fr/SNDS/Accueil).

A robust algorithm was built using the International Classification of Diseases (ICD-10) codes to identify COPD exacerbations admitted to any hospital in France over the last 10 years[2]. Precisely, Molinari et al[3] previously described the ICD-10 codes to select mild/moderate admitted COPD patients (ward admissions, with or without signs of acute respiratory failure, ARF), and moderately severe/severe admitted COPD patients (ICU admissions, with or without mechanical ventilation). The algorithm also encompasses the exclusion of specific ICD-10 codes, namely: asthma (J45); Status asthmaticus (J46); Bronchiectasis (J47); Postprocedural respiratory disorders, not elsewhere classified (J95); Other respiratory disorders (J98); Restrictive chronic respiratory failure (J96.1+1). Relevant data on demographics and comorbidities were analyzed to confirm that patients' profiles are in line with what can be expected in a COPD population.

An interrupted time series analysis (ITSA) was performed to model the impact of preventive measure implementation on hospital admissions considering three periods (before, during and after the COVID pandemic), and linear regression for graphical representation only. Specifically, the first period ('Before COVID pandemic') ranges from 2012 until March 2020; the second period ('COVID pandemic') corresponds to the COVID pandemic period from March 2020 to March 2022, comprising the official implementation and revocation of transmission prevention measures. Finally, the third period ('After COVID pandemic') starts in March 2022 until July 2023. The ITSA was based on Type II Sum Squares, ANCOVA, Lagged-Dependent Variable interrupted times series model, and was performed using the R package its.analysis that includes a bootstrap model, with 1000 replications, to estimate the 95% CI bootstrap, and adjusted F-value[4].

We performed 2-sided statistical tests with significance levels of 0.05. All statistical analyses were performed with R software (version 4.3.1.).

© 2024 Bourdin A et al. *JAMA Network Open.*

Ethical statement

According to French Law (Law 88-1138 relative to Biomedical Research of December 20, 1988, modified on August 9, 2004), this anonymous retrospective observational database study did not require approval by an ethics committee or informed signed consent from patients.

eReferences

1. Boudemaghe T, Belhadj I. Data Resource Profile: The French National Uniform Hospital Discharge Data Set Database (PMSI). *Int J Epidemiol.* 2017;46(2):392-392d.
2. Jouneau S, Dres M, Guerder A, et al. Management of acute exacerbations of chronic obstructive pulmonary disease (COPD). Guidelines from the Société de pneumologie de langue française (summary). *Revue des maladies respiratoires.* 2017;34(4):282-322.
3. Molinari N, Chanez P, Roche N, Ahmed E, Vachier I, Bourdin A. Rising total costs and mortality rates associated with admissions due to COPD exacerbations. *Respir Res.* 2016;17(1):149.
4. English P. The its.analysis R Package – Modelling Short Time Series Data. *SSRN Electron J.* 2019.

© 2024 Bourdin A et al. *JAMA Network Open.*
