# Supplementary material for: Hospitalizations for Chronic Obstructive Pulmonary Disease Exacerbation During COVID-19
Source: JAMA Netw Open. 2024 May 21;7(5):e2412383. doi: 10.1001/jamanetworkopen.2024.12383 (PMC11109769; doi:10.1001/jamanetworkopen.2024.12383)
Supplement: Supplement 2. — Data Sharing Statement [file jamanetwopen-e2412383-s002.pdf]

Data Sharing Statement

Bourdin. Hospitalizations for Chronic Obstructive Pulmonary Disease Exacerbation During COVID-19. *JAMA Netw Open*. Published May 21, 2024.
doi:10.1001/jamanetworkopen.2024.12383

Data
Data available: No

Additional Information
Explanation for why data not available: Confidential data from the national healthcare database (SNDS).
